# Iloprost-induced desensitization of the prostacyclin receptor in isolated rabbit lungs

**DOI:** 10.1186/1465-9921-8-4

**Published:** 2007-01-26

**Authors:** Ralph T Schermuly, Soni S Pullamsetti, Susanne C Breitenbach, Norbert Weissmann, Hossein A Ghofrani, Friedrich Grimminger, Sigrid M Nilius, Karsten Schrör, Jutta Meger-Kirchrath, Werner Seeger, Frank Rose

**Affiliations:** 1 University of Giessen Lung Center (UGLC), Medical Clinic II/V, Justus-Liebig-University Giessen, 35392 Giessen, Germany; 2 Institut fuer Pharmakologie und Klinische Pharmakologie, Heinrich-Heine-Universitaet Duesseldorf, 40225 Duesseldorf, Germany

## Abstract

**Background:**

The rapid desensitization of the human prostacyclin (IP) in response to agonist binding has been shown in cell culture. Phosphorylation of the IP receptor by protein kinase C (PKC) has been suggested to be involved in this process.

**Methods and results:**

In this study we investigated the vasodilatory effects of iloprost, a stable prostacyclin analogue, in perfused rabbit lungs. Continuous infusion of the thromboxane mimetic U46619 was employed to establish stable pulmonary hypertension. A complete loss of the vasodilatory response to iloprost was observed in experiments with continuous iloprost perfusion, maintaining the intravascular concentration of this prostanoid over a 180 min period. When lungs under chronic iloprost infusion were acutely challenged with inhaled iloprost, a corresponding complete loss of vasoreactivity was observed. This desensitization was not dependent on upregulation of cAMP-specific phosphodiesterases or changes in adenylate cyclase activity, as suggested by unaltered dose-response curves to agents directly affecting these enzymes. Application of a prostaglandin E1 receptor antagonist 6-isopropoxy-9-oxoxanthene-2-carboxylic acid (AH 6809) or the PKC inhibitor bisindolylmaleimide I (BIM) enhanced the vasodilatory response to infused iloprost and partially prevented tachyphylaxis.

**Conclusion:**

A three-hour infusion of iloprost in pulmonary hypertensive rabbit lungs results in complete loss of the lung vasodilatory response to this prostanoid. This rapid desensitization is apparently not linked to changes in adenylate cyclase and phosphodiesterase activation, but may involve PKC function and co-stimulation of the EP1 receptor in addition to the IP receptor by this prostacyclin analogue.

## Background

Prostacyclin (PGI_2_) is the major product of cyclooxygenases (COX) in the vascular endothelium and mediates potent anti-platelet, vasodilator, and anti-inflammatory actions by a prostacyclin receptor (IP) [1]. This receptor is a member of the G protein-coupled receptor (GPCR) superfamily and is coupled to adenylate cyclase (AC) and phospholipase C (PLC) [2-4]. The prostanoid receptors are classified into DP, IP, EP (EP 1–4), FP and TP receptors [5-7] with different affinities for agonists and different roles in signal transduction. The IP, EP2, EP4 and DP receptors are coupled to stimulation of adenylate cyclase, while the TP, EP1 and FP receptors are coupled to Ca^2+ ^mobilization. The EP3 receptor is an alternative spliced gene, with at least 8 isoforms identified so far. Depending on the subtype, this receptor can be negatively or positively coupled to Gs [8,9]. Agonist (PGI_2 _or PGI_2 _mimetics such as cicaprost, iloprost, carbacyclin, and prostaglandin E1)-binding to the IP receptor leads to activation of protein kinase A (PKA) by cyclic adenosine monophosphate (cAMP) [10]. Although, the above mentioned agonist specificities for IP receptors vary and none of them were highly selective for IP receptors alone. The affinity of cicaprost for human IP receptors is merely 3-fold higher than for the prostaglandin E2 EP4 subtype of receptor, which also couples to Gs [11], and only 17-fold higher than for the EP3 receptor in the mouse [12]. Iloprost is equipotent at both human and mouse IP and EP1 receptors, and carbacyclin and prostaglandin E1 show even greater affinity for EP3 than for IP receptors [11,12].

Disturbances to PGI_2 _synthesis [13,14], as well as polymorphisms of the PGI_2_-synthase (PGIS) [15], have been linked to severe pulmonary hypertension. Substitution of PGI_2_, either by overexpression of the PGIS [16] in an experimental model or by application of PGI_2 _[17,18] or its analogues iloprost [19,20] or beraprost [21,22] in patients decreases pulmonary artery pressure. However, tolerance of the lung vasodilatory response to continuously infused PGI_2 _rapidly develops in patients with severe pulmonary hypertension, and dose adjustments have to be made [17,23]. In COPD patients with pulmonary hypertension, the pulmonary vasodilatory response to continuously infused PGI_2 _was found to dissipate within 24 h [24]. Marked tolerance to the anti-mitogenic actions of PGI_2 _developed within 24 h in coronary artery smooth muscle cells [25]. Basic studies demonstrated that desensitization of the IP receptor occurs within minutes after exposure to agonists and is due to agonist-induced receptor phosphorylation, mainly mediated by PKC [26], with subsequent sequestration of the intact receptor and removal from the cell surface [26,27]. In addition, there is evidence for changes in adenylate cyclase and phosphodiesterase (PDE) activation occurring in response to IP receptor stimulation, which may contribute to loss of the vasodilatory response to PGI_2 _and its analogues [28-30]. In the present study, we employed the model of pulmonary hypertensive perfused rabbit lungs to investigate the dose-response relationship and features of tolerance development upon continuous iloprost infusion in the intact lung vasculature.

## Materials and methods

### Materials

Sterile Krebs-Henseleit buffer (KHB) was obtained from Serag-Wiessner (Naila, Germany). The thromboxane-A_2 _mimetic U46619 was supplied by Paesel-Lorei (Frankfurt, Germany) and iloprost by Schering (Berlin, Germany). All other chemicals were purchased from Merck (Darmstadt, Germany).

### Isolated lung model

The perfused rabbit lung model has previously been described in detail [31]. Briefly, rabbits of either sex weighing 2.6 to 2.9 kg were anticoagulated with heparin (1000 U/kg) and anaesthetized with intravenous ketamine/xylazine. Tracheostomy was performed and the animals were ventilated with room air. After mid-sternal thoracotomy, catheters were placed into the pulmonary artery and the left atrium, and perfusion with KHB was started. For washout of blood, perfusate was initially not recirculated and the lungs were removed without interruption of ventilation and perfusion. The lungs were placed in a temperature-equilibrated chamber at 37.5°C, freely suspended from a force transducer for monitoring of organ weight. In a recirculating system the flow was slowly increased to 120 ml/min (total volume 350 ml). Left atrial pressure was set at 2 mmHg in all experiments. In parallel with onset of artificial perfusion, an air mixture of 80.5% N_2_, 15% O_2 _and 4.5% CO_2 _was used for ventilation. Tidal volume (11 ml/kg) and frequency (10 – 13 breaths/min) were adapted to maintain the pH of the recirculating buffer in the range between 7.35 and 7.37. A positive end-expiratory pressure of 1 mmHg was used throughout. The PO_2 _and PCO_2 _values in the post-lung buffer fluid ranged between 100 and 120 mmHg and 38 and 43 mmHg, respectively. Pressures in the pulmonary artery, the left atrium and the trachea were registered by means of small diameter tubing threaded into the perfusion catheters and the trachea and connected to pressure transducers (zero referenced at the hilum). The whole system was heated to 37.5°C.

Lungs included in the study had 1) a homogeneous white appearance with no signs of hemostasis, edema or atelectasis; 2) pulmonary artery and ventilation pressures in the normal range; and were 3) isogravimetric (lung weight gain < 0.2 g/h) during an initial steady state period of at least 45–60 min.

### Experimental protocols

#### Isolated lung experiments

As described previously [32], a sustained increase of P_PA _from ≈6 to ≈24 mmHg was achieved by continuous infusion of the thromboxane-mimetic U46619 with a dose range of 70–160 pmol·kg^-1^·min^-1^. Individual titration was performed. This level of pulmonary hypertension was then maintained for at least 320 min with variations in P_PA _of less than 2 mmHg. The following experimental groups were employed:

*Control lungs (n = 4): *No interventions were undertaken.

*U46619-infused lungs (n = 4): *After termination of the steady state period, U46619 was continuously infused for 320 min to provoke an increase in P_PA _to ~24 mmHg.

*Iloprost infusion (n = 4): *U46619 was administered as described. 20 min after onset of U46619 infusion, iloprost was applied by a bolus injection of 200 ng, followed by a continuous infusion of 33 ng/kg/h to achieve stable buffer concentrations in the range of 350 pg/ml.

*Intravenous PGI_2 _application (n = 4): *U-46619 was administered as described. 20 min after onset of U-46619 infusion, PGI_2 _was continuously applied at a dose of 50 ng· kg^-1^·min^-1^ until termination of the experiments.

*Rolipram i.v. (n = 4): *After adjusting stable pulmonary hypertension, the PDE4 inhibitor rolipram was applied in increasing doses (dose range 0.001–10 μM).

*Iloprost infusion and rolipram i.v. (n = 4): *Iloprost was applied by a bolus injection of 200 ng, followed by a continuous infusion of 33 ng/kg/h. 240 min after starting the iloprost infusion, rolipram was applied in increasing doses (dose range 0.001–10 μM).

*Motapizone i.v. (n = 4): *After adjusting stable pulmonary hypertension, the PDE3 inhibitor motapizone was applied in increasing doses (dose range 0.01–100 μM).

*Iloprost infusion and motapizone i.v. (n = 4): *Iloprost was applied by a bolus injection of 200 ng, followed by a continuous infusion of 33 ng/kg/h. 240 min after starting the iloprost infusion, motapizone was applied in increasing doses (dose range 0.01–100 μM).

*Iloprost inhalation (n = 4): *When stable pulmonary hypertension was achieved, iloprost was nebulized (deposited dose 75 ng) by means of an ultrasonic nebulizer as described previously [33,34].

*Iloprost infusion and iloprost inhalation (n = 4): *U46619 was continuously infused and iloprost was applied by a bolus injection of 200 ng, followed by a continuous infusion of 33 ng/kg/h. 240 min after starting the iloprost infusion, iloprost was nebulized (deposited dose 75 ng).

*Forskolin i.v. (n = 4): *After adjusting stable pulmonary hypertension, the adenylate cyclase stimulator forskolin was applied in increasing doses (dose range 10–100 μM).

*Iloprost infusion and forskolin i.v. (n = 4): *Iloprost was applied by a bolus injection of 200 ng, followed by a continuous infusion of 33 ng/kg/h. 240 min after starting the iloprost infusion, forskolin was applied in increasing doses (dose range 10–100 μM).

*Bisindolylmaleimide I (BIM) infusion (n = 4): *BIM (1 μM) was applied by a bolus injection after stable adjustment of pulmonary hypertension with U46619. As described previously, stable buffer levels of BIM are achieved by a single application in the setup of isolated perfused rabbit lungs [35].

*Iloprost infusion in presence of BIM (n = 4): *U46619 was administered as described. After stable adjustment of pulmonary hypertension, BIM (1 μM) was applied by a bolus injection. Iloprost was bolus injected and infused as described above.

*6-isopropoxy-9-oxoxanthene-2-carboxylic acid (AH 6809) infusion (n = 4): *AH 6809 (3 μM) was applied by a bolus injection after stable adjustment of pulmonary hypertension with U46619. As described previously, AH6809 demonstrated long acting EP1 blocking activity in fluid perfused small intestine segments after a single application [36].

*Iloprost infusion in presence of AH 6809 (n = 4): *U46619 was administered as described. 45 min after onset of U46619 infusion, AH 6809 (3 μM) was applied as a bolus injection and the bolus injection plus infusion of iloprost was started as in the preceding groups.

### Cell culture

Rabbit pulmonary smooth muscle cells (PSMC) were prepared as described. Primary SMC were isolated from rabbit pulmonary artery by carefully preparing <1 mm^3^ pieces of media, devoid of adventitial tissue as assessed by microscopic control. The pieces of media were placed into 12-well cell culture plates with 500 μl culture medium. The isolated pulmonary smooth muscle cells (PSMC) identity was verified by characteristic appearance in phase-contrast microscopy, indirect immunofluorescent antibody staining for smooth muscle-specific isoforms of α-actin and myosin (at least 95% of cells stained positive), and lack of staining for von Willebrand factor and vimentin, indicating that the cultures did not contain significant numbers of endothelial cells or adventitial fibroblasts [37]. Smooth muscle cells were grown in Dulbecco's modified Eagle's high glucose medium supplemented with 10% fetal bovine serum, 100 μg/ml streptomycin and 100 units/ml penicillin.

### Desensitization assays and cAMP measurement

Smooth muscle cells were grown to 90% confluence in 24-well plates, as previously described. For desensitization assays, cells were treated with iloprost for the indicated times. Then, cells were washed three times with HBSS and preincubated in HBSS containing 1 mg/ml BSA, 10 mM HEPES (pH 7.3) and 1 mM IBMX for 10 min at 37°C. Afterwards cells were again stimulated with iloprost (100 nM) or forskolin (10 μM) for 10 min and cAMP measurements were performed as described below. Reactions were stopped by aspiration and addition of ice-cold 96% ethanol. Dried samples were overlaid with 300 μl RIA-buffer (150 mM NaCl, 8 mM Na_2_HPO_4_, 2 mM NaH_2_PO_4_, pH 7.4) and frozen overnight at -80°C. cAMP in the supernatant was determined by radioimmunoassay [Steiner et al., 1972]. Protein determination was performed according to the method of Bradford. Cells treated with vehicle alone (no ligand) or forskolin (50 μM) were served as negative and positive controls respectively for the cAMP measurement.

### Data analysis

Values are means ± SEM. Student's t-test was performed for comparison of two values. For multiple comparisons, ANOVA was followed by post hoc test (Bonferroni/Dunn method). Statistical significance was considered at p < 0.05.

## Results

### Baseline conditions

After termination of the steady state period, all lungs displayed P_PA _values in the range between 5 and 7 mmHg.

### U46619-elicited pulmonary hypertension

Continuous infusion of U46619 provoked an increase in P_PA _to 23.4 ± 0.9 mmHg within 25 min, with subsequent plateau of the pulmonary hypertension. This level of pulmonary hypertension was then maintained for at least 300 min with variations in P_PA _of less than 2 mmHg. Total weight gain at the end of the experiments was 6.5 ± 2.7 g.

### U46619-elicited pulmonary hypertension and infusion of iloprost or PGI_2_

**Figure 1:**
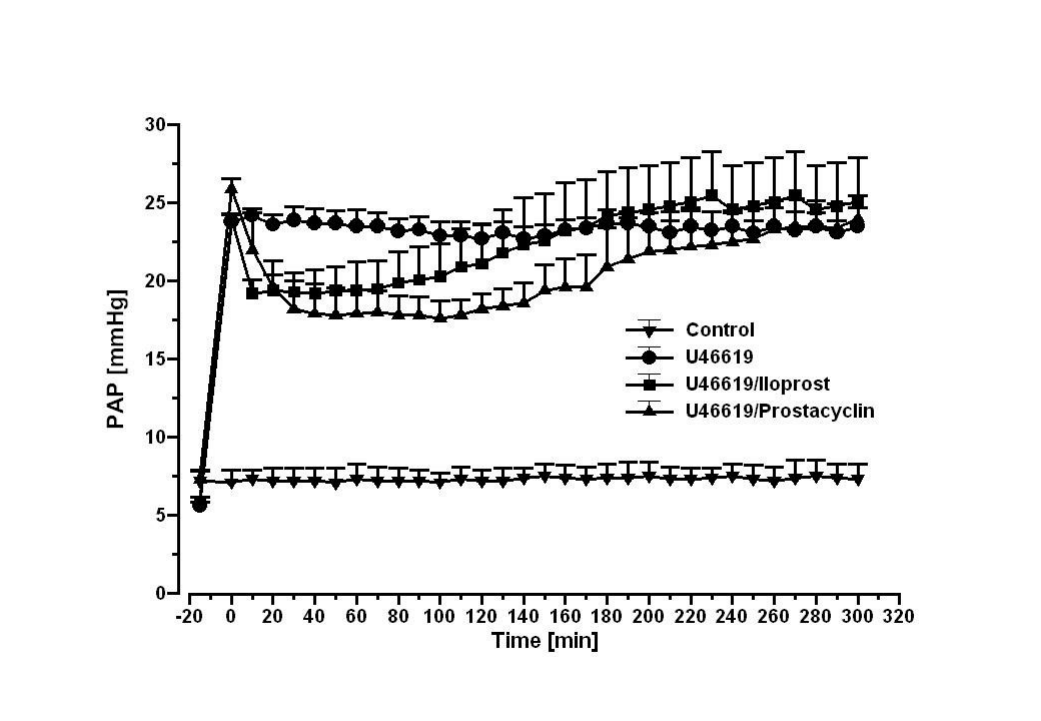
**Prostacyclin receptor (IP) desensitization in U46619-induced pulmonary hypertension in intact rabbit lungs in response to PGI_2 _or iloprost infusion**. Pulmonary hypertension was adjusted by continuous infusion of the thromboxane mimetic U46619. After a bolus injection of 200 ng iloprost followed by an infusion of 33 ng iloprost per hour (stable buffer levels of ~350 pg/ml) or continuous infusion of PGI_2 _(50 ng/min) is administered and measured their influence on U46619-elicited pulmonary hypertension.

As in the preceding group, U46619 infusion for establishing stable pulmonary hypertension was performed. Bolus injection of 200 ng iloprost was followed by an infusion of 33 ng iloprost per hour. This procedure resulted in a significant vasodilatory response with P_PA _values decreasing by a mean of 3.3 mmHg. Similarly, the continuous infusion of a more selective IP receptor ligand, PGI_2 _also resulted in significant vasodilatory responses (Fig 1). Stable iloprost buffer concentrations in the range of 350–380 pg/ml were documented over the entire perfusion period. A slow increase in P_PA _toward pre-infusion values was noted to commence after 100–120 min, and 200 min after beginning of the iloprost infusion, the vasodilatory effect was virtually fully lost. The total weight gain of the lungs was 5.3 ± 3.2 g, and thus did not differ from the groups with mono U46619 infusion.

### Dose-effect curves of PDE-inhibitors with or without infusion of iloprost

**Figure 2:**
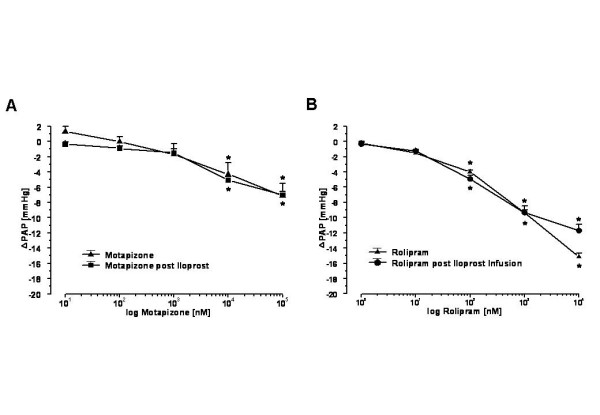
**Dose effect curve of the PDE3 inhibitor motapizone or PDE4 inhibitor rolipram on U46619-elevated pulmonary artery pressure in untreated and desensitized (post iloprost infusion) lungs**. Motapizone and rolipram dose-dependently reduced the elevated pulmonary artery pressure. There was no change in the dose response curve when applying identical doses of motapizone 240 min after iloprost infusion. (A) The decrease in pulmonary artery pressure (ΔPAP, as decrease of U46619-induced pulmonary hypertension) in response to motapizone or (B) rolipram (concentrations related to the recirculating perfusate) is given. (mean ± SEM of four independent experiments each; SEM bars are not included when smaller than symbol). *: p < 0.05 as compared to U46619-elevated pulmonary artery pressure.

As shown in Fig. 2A, the intravascular administered PDE3 inhibitor motapizone (dose range 0.01–100 μM) effected a dose-dependent reduction of the elevated P_PA _values in lungs with U46619-elicited pulmonary hypertension. There was no shift in the dose response curve when applying identical doses of motapizone 240 min after onset of iloprost infusion. Similar experiments were performed with the PDE4 inhibitor rolipram, which dose-dependently reduced pulmonary artery pressure in U46619 preconstricted lungs, with identical dose-effect curves in the absence and presence of iloprost infusion (Fig. 2B). Total weight gain was 5.7 ± 2.9 g and 5.3 ± 1.9 g (iloprost infusion) in the motapizone treated lungs and 4.6 ± 2.2 g and 5.0 ± 2.6 g (iloprost infusion) in the rolipram treated lungs, respectively.

### Nebulization of iloprost

**Figure 3:**
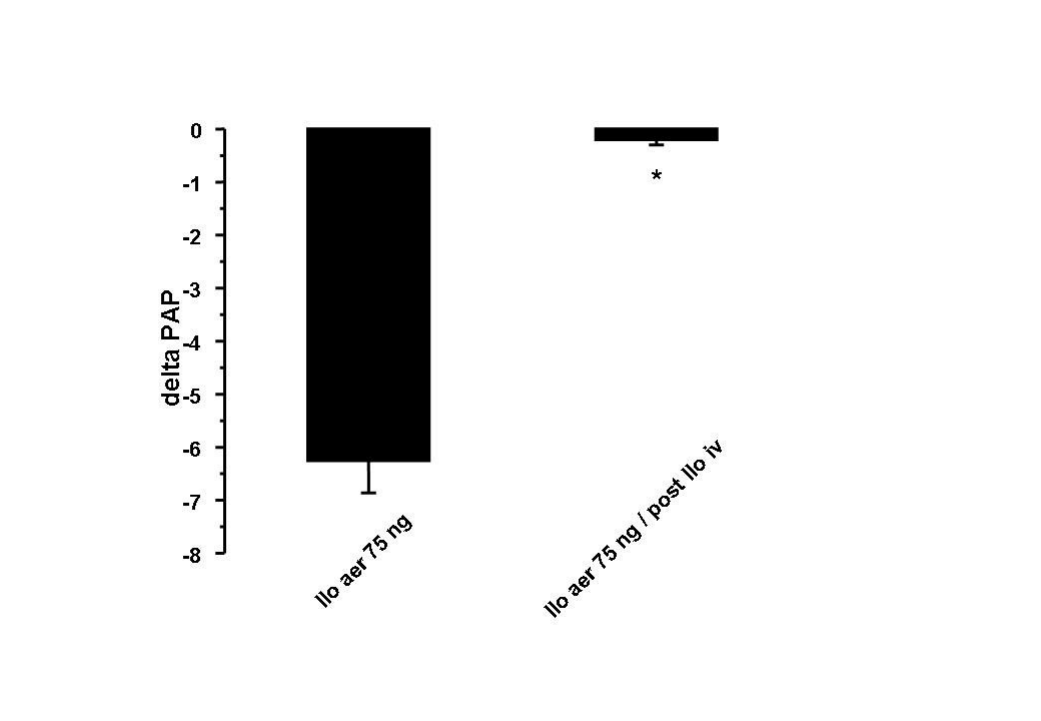
**Effect of inhaled iloprost on U46619-elevated pulmonary artery pressure in untreated and desensitized (post iloprost infusion) lungs**. Inhalation of aerosolized iloprost for 10 min (deposited dose 75 ng) resulted in a significant reduction of the U46619-induced pulmonary hypertension, with PAP values decreasing by a mean of 6.3 mmHg. The vasodilatory potency of inhaled iloprost was completely lost in the desensitization group (iloprost infusion for 240 min). *: p < 0.05 as compared to U46619-elevated pulmonary artery pressure.

Inhalation of aerosolized iloprost (total dose 75 ng inhaled over 10 min) resulted in a significant reduction of the U46619-induced pulmonary hypertension, with PAP values decreasing by a mean of 6.3 mmHg (Fig. 3). In separate experiments, iloprost was infused in advance for 240 min, and then nebulization was started. There was virtually no response to inhaled iloprost after this preceding iloprost infusion period. The total weight gain of the lungs was 6.7 ± 2.9 g at the end of experiments.

### Dose-effect curves of forskolin

**Figure 4:**
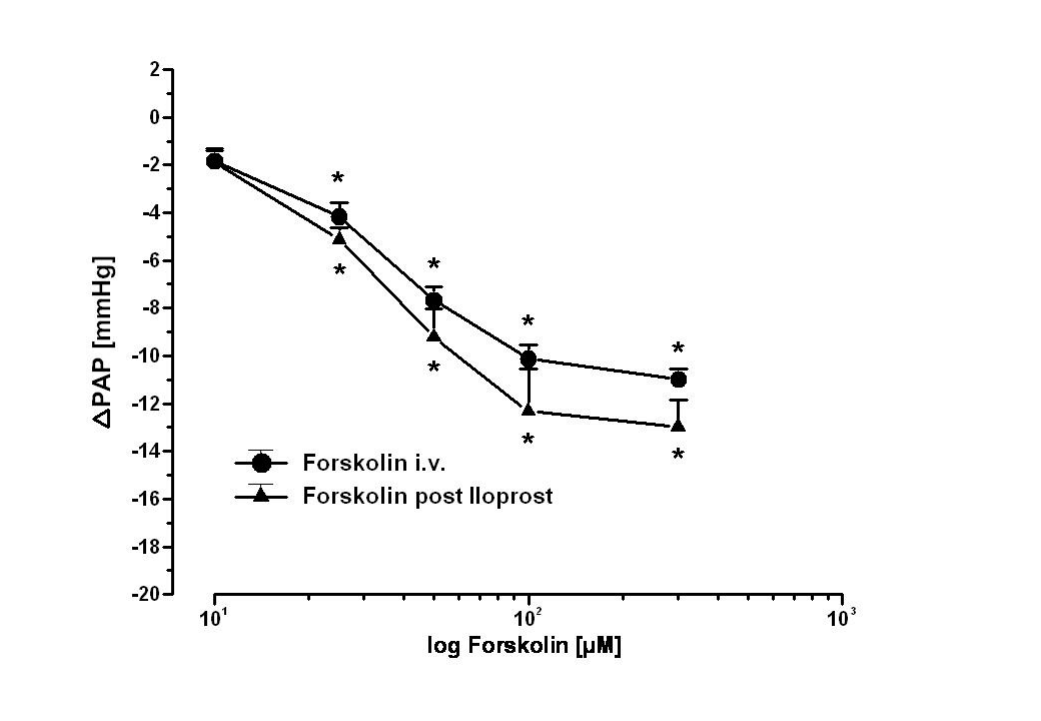
**Dose effect curve of the adenylate cyclase activator forskolin on U46619-elevated pulmonary artery pressure in untreated and desensitized (post iloprost infusion) lungs**. Forskolin dose-dependently reduced the elevated pulmonary artery pressure. There was no change in the dose response curve when applying identical doses of forskolin 240 min after iloprost infusion. The decrease in pulmonary artery pressure (ΔPAP, as decrease of U46619-induced pulmonary hypertension) in response to forskolin (concentrations related to the recirculating perfusate) is given (mean ± SEM of 4 independent experiments each; SEM bars are not included when smaller than symbol). *: p < 0.05 as compared to U46619-elevated pulmonary artery pressure.

As shown in Fig. 4, the adenylate cyclase activator forskolin (dose range 10–300 μM) reduced the U46619-elicited pulmonary hypertension in a dose dependant manner. After iloprost infusion for 240 min, an identical dose response curve was observed. Total weight gain was 5.6 ± 2.1 g at the end of the experiments.

### Infusion of iloprost: effects of BIM

**Figure 5:**
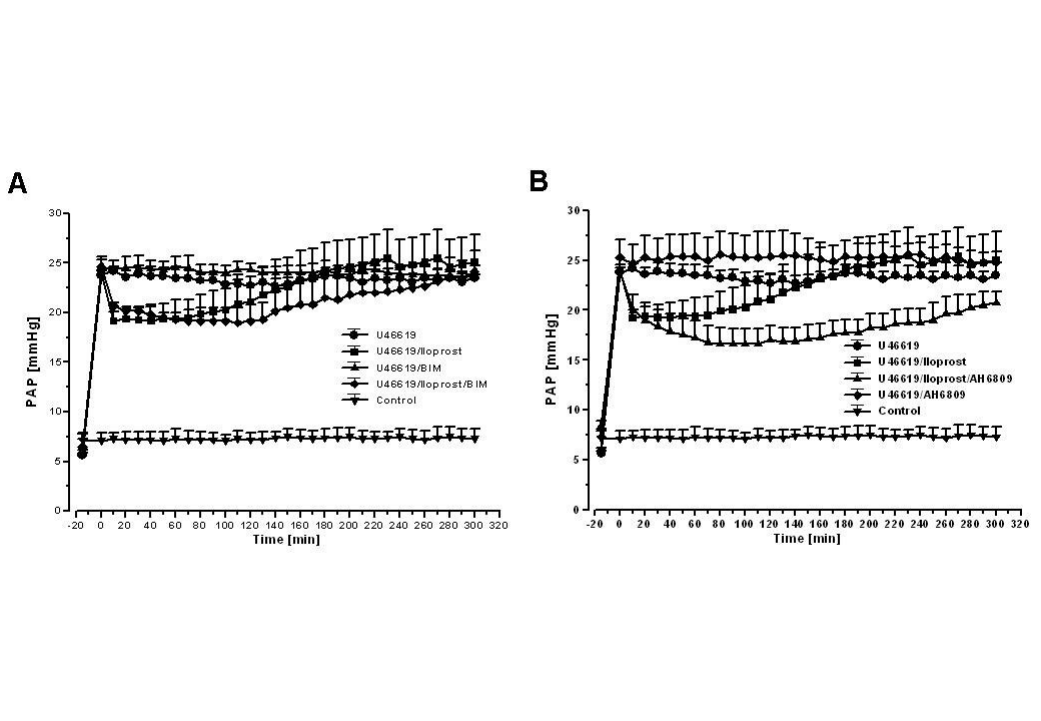
**Influence of iloprost infusion and its combination with AH 6809 and BIM on U46619-elicited pulmonary hypertension**. Pulmonary artery pressure (PAP) is given (mean ± SEM of 4 independent experiments each, SEM bars are missing when enclosed by the symbol). (A) The prostaglandin E_1 _receptor antagonist AH 6809 alone or in combination with iloprost (pre-applied followed by an iloprost infusion). (B) The PKC inhibitor BIM alone or in combination with iloprost (pre-applied followed by an iloprost infusion).

**Figure 6:**
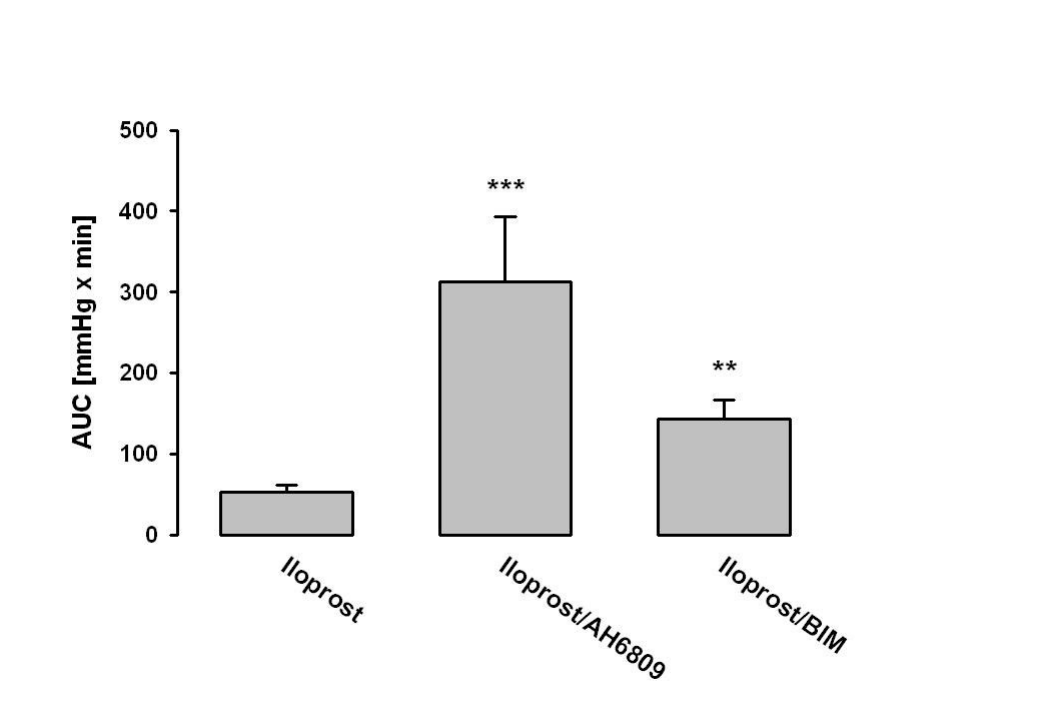
**Influence of iloprost infusion and its combination with AH 6809 and BIM on the area under the curve of the U46619-elicited pulmonary hypertension**. Area under the curve (AUC) is given (mean ± SEM of 4 independent experiments each). The AUC was calculated by standard techniques and is given as mmHg × min. **: p < 0.01; ***: p < 0.001 as compared to AUC of sole iloprost infusion.

As in the preceding groups, U46619 infusion for establishing stable pulmonary hypertension was performed. Subsequently, the PKC inhibitor BIM at a dose of 1 μM was applied alone or in combination with iloprost. In combination group, after 5 min of BIM application, bolus injection of 200 ng iloprost was followed by an infusion of 33 ng iloprost per hour. This procedure resulted in a significant vasodilatory response with P_PA _values decreasing by a mean of 4.3 mmHg (Fig. 5A). However, the sole BIM application demonstrated no effects on U46619-elicited pulmonary hypertension (Fig. 5A). The calculated area under the curve (AUC) of the pressure curve was significantly increased as compared to sole iloprost infusion (Fig. 6). The loss of the vasodilatory response to iloprost was somewhat retarded, but not prevented, as compared to single iloprost application. The total weight gain of the lungs was 5.8 ± 2.5 g.

### Infusion of iloprost: effects of AH 6809

After adjusting stable pulmonary hypertension, the EP1 receptor antagonist AH 6809 at a dose of 3 μM was applied alone or in combination with iloprost, without effecting pulmonary artery pressure. In combination group, after 5 min of AH 6809 application, a bolus injection of 200 ng iloprost was followed by an infusion of 33 ng iloprost per hour. The maximum vasodilatory response to iloprost was 11.4 mmHg (Fig. 5B) and the area under the curve (AUC) of the pressure curve was 305 mmHg × min (p < 0.05 versus sole iloprost infusion) (Fig. 6). The total weight gain of the lungs was 4.8 ± 2.0 g. In contrary, the AH 6809 application alone had no effect on U46619-induced tension (Fig. 5B).

### Measurement of cyclic AMP

**Figure 7:**
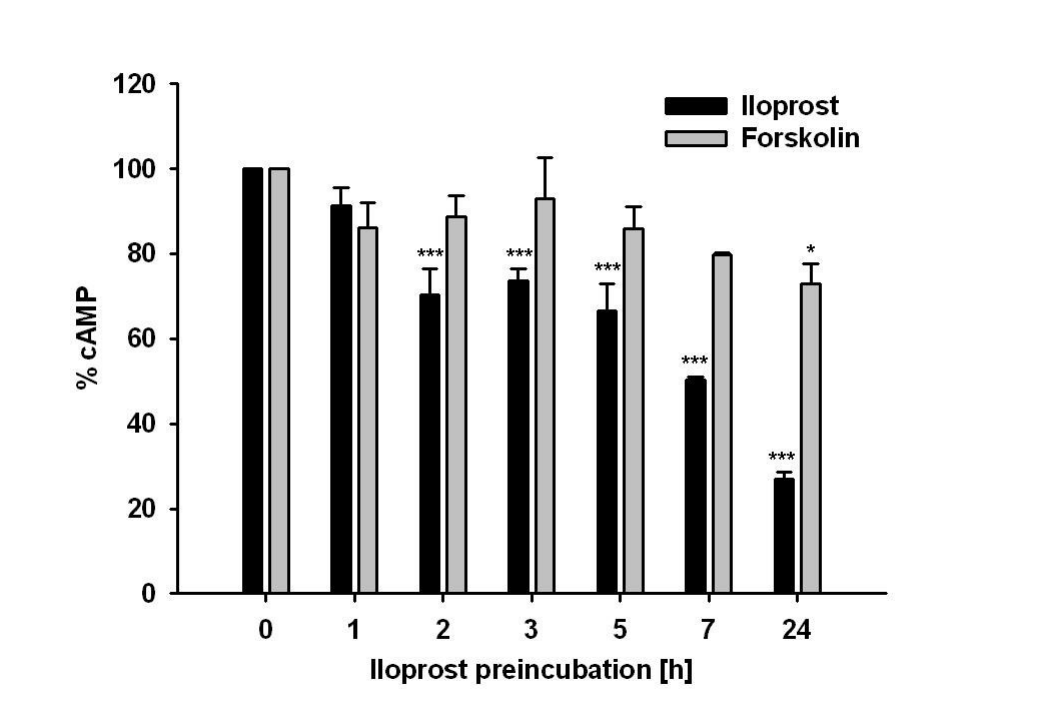
**Prostacyclin receptor (IP) desensitization in rabbit pulmonary smooth muscle cells**. Cells were continuously stimulated with 100 nM iloprost for the indicated preincubation times. cAMP response to iloprost (100 nM) or forskolin (10 μM) was assayed in these cells and is given as a percent of the non-desensitized control. Values are expressed as mean ± SEM of three independent experiments performed in triplicate. *: p < 0.05; ***: p < 0.001 as compared to non-desensitized control.

We assayed the desensitization kinetics of iloprost and forskolin in rabbit pulmonary smooth muscle cells (Fig. 7). Incubation of cells with iloprost led to a time-dependent reduction of iloprost induced cAMP formation to 88% (1 h), 71% (2 h), 76% (3 h), 66% (5 h), 54% (7 h) and 26% (24 h) of control. Incubation of cells with iloprost and stimulation of cells with forskolin led to a reduction of cAMP formation to 83% (1 h), 84% (2 h), 91% (3 h), 83% (5 h), 79% (7 h) and 75% (24 h) of control.

## Discussion

The desensitization of the IP receptor after incubation of platelets [38,39] or IP receptor overexpressing cells [26,27,40,41] with PGI_2 _or its analogues in vitro is a well-known phenomenon. Tolerance development of the lung vasodilatory response to PGI_2 _has also been observed in patients with severe pulmonary hypertension being chronically treated by intravenous infusion of the prostanoid [23,24]. In the present study we show that such desensitization occurs within 180 min in perfused rabbit lungs.

### Desensitization is not dependant on increased PDE activity

Phosphodiesterases (PDE) are enzymes that hydrolyze cyclic AMP and cyclic GMP, the second messengers of PGI_2_, and NO [42,43]. The characterization of the various PDEs currently known has profited from the employment of selective PDE inhibitors. Concerning the lung vasculature, the presence of the PDE isoenzymes 1, 3, 4 and 5 in the cytosolic and particulate phases (homogenized human pulmonary artery tissue) has been demonstrated [44]. There is strong evidence of PDE activation and upregulation in response to intracellular elevation of cAMP. It has been shown recently that cyclic AMP upregulates isoforms of PDE4 in airway smooth muscle cells [45], monocytes [46], endothelial cells [47] and Jurkat T-cells [48]. In addition, short term activation (phosphorylation) of PDE4 via PKA was demonstrated by treatment of cells with forskolin, a stimulator of adenylate cyclase [49]. Furthermore, treatment of rats with 7-oxo-prostacyclin resulted in a significant increase in PDE4 activity, which attenuated the elevation of isoprenaline-induced cAMP levels as well as the contractile force development in a Langedorff-heart preparation [50]. Similar results were obtained for PDE3 which has been shown to be regulated on the level of activity [48] and expression [32] by increased cAMP levels. In the current study, we employed motapizone and rolipram, selective inhibitors of PDE3 and PDE4, respectively and performed dose response studies on vasodilation in normal and desensitized (iloprost-treated) isolated rabbit lungs. As reported earlier, these PDE inhibitors are potent pulmonary vasodilators [51] and we found no shift in the dose response curve in desensitized lungs. Thus, there is no evidence of increased PDE activity in iloprost-treated isolated rabbit lungs.

### Desensitization is not dependant on adenylate cyclase activity

We employed forskolin as direct activator of adenylate cyclase and performed dose response studies on pulmonary vasodilation in isolated rabbit lungs. The response to forskolin in the desensitized (iloprost-treated) organs remained intact (and seemed to be slightly increased), indicating no regulation of adenylate cyclase. Similar results were obtained in rats chronically treated with albuterol in which acetylcholine (ACh)-induced bronchoconstriction was reversible by forskolin, but not by albuterol itself, suggesting that the intracellular site of desensitization is upstream of adenylate cyclase [27].

Moreover, the cAMP production of iloprost-pretreated rabbit pulmonary smooth muscle cells in response to forskolin was also not significantly reduced, except in 24 hours of iloprost-pretreated group (to 75% of control). This is quite contrary to iloprost responses, where a significantly reduced cAMP levels (to 25% of control) was observed, thereby excluding the down-regulation of adenylate cyclase as a possible reason for reduced cAMP formation after continuous stimulation of cells with iloprost. In this context, desensitization of IP receptors by iloprost may possibly explained by its direct influence on the receptor, a mechanism upstream of adenylate cyclases. This involves rapid receptor phosphorylation, which results in uncoupling of receptor-G protein interactions and subsequent receptor internalization. These events result in a diminished response to agonist [26]. This may also be due to preferential reduction of domain-bound pool of the cognate Gproteins GsαL and GsαS [52].

On the other hand, iloprost induced vasorelaxation responses may also be via cAMP-independent manner. Consistent with the observation, PGI_2 _analogues iloprost were shown to activate MaxiK and K_IR _channel and subsequent vasorelaxation through a cAMP-independent, G(s)-protein mediated mechanism in vascular smooth muscle cells [53,54]. Concomitantly in guinea-pig aorta, cyclic AMP-dependent and -independent pathways were recently proposed to underlie the relaxation induced by the PGI_2 _analogues, iloprost and Beraprost [55-57]. In contrast, in rat tail artery, it is thought that channel phosphorylation by cyclic AMP-dependent PKA is responsible for the MaxiK channel-mediated vascular relaxation due to IP receptor stimulation by iloprost [58,59]. This is well in line with investigations in human fibroblasts, where cells were challenged at any time point of iloprost pre-treatment with forskolin and fully maintained their cAMP response [30]. In contrast, Sobolewski et al., demonstrated in rat pulmonary smooth muscle cells (PSMCs) which were exposed to cicaprost, another PGI_2 _analogue, a significant reduction in forskolin-induced cAMP production [60] as well as a decrease in adenylate cyclase isoform 2 and 5/6 expression after one hour of exposure to this prostanoid. However, both isoforms were expressed at control levels when PSMCs were incubated for longer time periods with cicaprost. It is possible that the agonists employed, as well as species differences are responsible for these conflicting data.

### Iloprost influences the EP1 receptor in addition to the IP receptor

It is well known that iloprost, the clinically approved compound for treatment of thromboangiitis obliterans [20] and pulmonary arterial hypertension [5], is not highly selective for the IP receptor but also activates the EP1receptor which is coupled to Gq and signals via IP_3 _generation and increased intracellular calcium (Ca^2+^) [61]. To investigate the effects of possible involvement of the EP1receptor we employed the EP1receptor antagonist AH 6809 and found a dramatic increase in the efficacy of intravenous iloprost. In contrary, the AH 6809 application alone had no effect on U46619-induced tension. Thus, the agonistic effects on EP1receptor explain the differences between iloprost and selective IP agonists in inhibition of smooth muscle cell response [26,40].

### PKC inhibition and IP receptor desensitization

There is evidence that the initial desensitization process is dependent on phosphorylation of the IP receptor by PKC [41,62]. After phosphorylation, the IP receptor is sequestered to clathrin-coated vesicles and by a dynamin-dependant process internalized into endosomes [41]. This receptor sequestration is, unlike desensitization, PKC-independent [27] and a time of approximately 18 h is needed for a resensitization in human fibroblasts [27,30]. However, several studies including our work employed inhibitors of PKC (GF-109203X, BIM) and could not or could only partially prevent receptor desensitization, suggesting a minor role of PKC in the desensitization process.

In conclusion, this study demonstrates for the first time in an intact lung model that the PGI_2 _analogue iloprost caused desensitization of the IP receptor within 2 to 3 hours, resulting in complete loss of responsiveness after this time period. Desensitization was not due to increased PDE activity or loss of adenylate cyclase activity, but may involve PKC function and co-stimulation of the EP1 receptor in addition to the IP receptor by this PGI_2 _analogue.
